# IS -94INS/DELATTG POLYMORPHISM IN THE NUCLEAR FACTOR KAPPA-B1 GENE (NFKB1) ASSOCIATED WITH NECROTIZING ENTEROCOLITIS?

**DOI:** 10.1590/0102-672020220002e1717

**Published:** 2023-01-09

**Authors:** Danielle Lopes Teixeira FERDINANDO, Fernanda Del Campo Braojos FRAGA, Vânia Belintani PIATTO, Antônio Soares SOUZA

**Affiliations:** 1 Faculty of Medicine of São José do Rio Preto, Pediatrics and Pediatric Surgery – São José do Rio Preto (SP), Brazil; 2 Faculty of Medicine of São José do Rio Preto, Image Unit – São José do Rio Preto (SP), Brazil; 3 Faculty of Medicine of São José do Rio Preto, Anatomy Unit – São José do Rio Preto (SP), Brazil.

**Keywords:** Enterocolitis, Necrotizing, Infant, Premature, NF-kappa B, Polymorphism, Genetic, Enterocolite Necrosante, Recém-Nascido Prematuro, NF-kappa B, Polimorfismo Genético

## Abstract

**BACKGROUND:**

Abnormalities in the different stages of the intestinal maturation process cause metabolic and molecular changes. Among the genetic alterations associated with necrotizing enterocolitis, the -94ins/delATTG polymorphism in *NFKB1* gene leads to unregulated activation of the NFKB protein due to an increase in the inherent pro-inflammatory state of the premature intestine.

**AIMS:**

To determine the prevalence of the -94ins/delATTG polymorphism in *NFKB1* gene in neonates with and without necrotizing enterocolitis.

**METHODS:**

This is a case-control study, in which 25 neonates were evaluated as the case group and 50 neonates as the control group, of both genders. DNA was extracted from peripheral blood leukocytes, and the site encompassing the polymorphism was amplified by molecular techniques (polymerase chain reaction/polymorphism in restriction fragment length).

**RESULTS:**

Necrotizing enterocolitis was diagnosed in 25 (33%) neonates and, of these, 3 (12%) died. Male gender was more prevalent in both groups (p=0.1613): cases (52%) and controls (62%). Moderate and extreme preterm newborns were predominant in both groups: cases (80%) and controls (88%) (p=0.3036). Low birth weight and extremely low birth weight newborns were the most prevalent in cases (78%), and very low birth weight and extremely low birth weight were the most prevalent in controls (81%) (p=0.1073). Clinical treatment was successful in 72%, and hospital discharge was achieved in 88% of newborns with NEC. The -94ins/delATTG polymorphism in *NFKB1* gene was not identified in all the 150 alleles analyzed (100%).

**CONCLUSIONS:**

The absence of the -94ins/delATTG polymorphism in *NFKB1* gene in newborns with and without necrotizing enterocolitis does not rule out the possibility of alterations in this and/or in other genes in newborns with this condition, which reinforces the need for further research.

## RESUMO

**RACIONAL:**

Anormalidades nas diferentes fases do processo de maturação intestinal causam alterações metabólicas e moleculares. Dentre as alterações genéticas associadas à enterocolite necrotizante, o polimorfismo -94ins/delATTG no gene NFKB1 leva à ativação desregulada da proteína NFKB devido ao aumento do estado pró-inflamatório inerente ao intestino prematuro.

**OBJETIVOS:**

Determinar a prevalência do polimorfismo -94ins/delATTG no gene NFKB1 em neonatos com e sem enterocolite necrotizante.

**MÉTODOS:**

Trata-se de um estudo caso-controle, no qual foram avaliados 25 neonatos como grupo caso e 50 neonatos como grupo controle, de ambos os sexos. O DNA foi extraído de leucócitos do sangue periférico e o sítio que engloba o polimorfismo foi amplificado por técnicas moleculares (reação em cadeia da polimerase/polimorfismo no comprimento do fragmento de restrição).

**RESULTADOS:**

Enterocolite necrosante foi diagnosticada em 25 (33%) neonatos e, destes, 3 (12%) foram a óbito. O gênero masculino foi mais prevalente em ambos os grupos (p=0,1613): casos (52%) e controles (62%). Os prematuros moderados e extremos foram predominantes em ambos os grupos: casos (80%) e controles (88%) (p=0,3036). Recém-nascidos de baixo peso e extremo baixo peso foram os mais prevalentes nos casos (78%) e de muito baixo peso e extremo baixo peso foram os mais prevalentes nos controles (81%) (p=0,1073). O tratamento clínico foi bem-sucedido em 72% e a alta hospitalar foi obtida em 88% dos recém-nascidos com enterocolite necrotizante. O polimorfismo -94ins/delATTG no gene NFKB1 não foi identificado em todos os 150 alelos analisados (100%).

**CONCLUSÕES:**

A ausência do polimorfismo -94ins/delATTG no gene NFKB1 em recém-nascidos com e sem enterocolite necrosante não afasta a possibilidade de alterações neste e/ou em outros genes em recém-nascidos com esta condição, o que reforça a necessidade de novas pesquisas.

## INTRODUCTION

Necrotizing enterocolitis (NEC) is one of the most common and unpredictable intestinal diseases involving systemic inflammatory responses in preterm infants. It affects 1–8% of all newborns (NB) admitted to the neonatal ICU, and its lethality is very high, ranging between 10% and 50%. In preterm NB weighing less than 1500 g, the incidence of NEC varies from 4% to 13%. Those born at term or near term can also be affected in 5–10% of classic cases of the disease^2,8,35^.

No specific pathogen is consistently associated with the emergence of enterocolitis. Although the exact cause is still considered an enigma, it is assumed that NEC results from an initial aggression to the intestinal mucosa through the action of a range of factors on an immature gastrointestinal system, followed by a series of cascading inflammatory reactions and proliferation and bacterial invasion of the intestinal mucosa, culminating in coagulation necrosis of the affected areas^11,14,29,30^.

Several genes, members of the NFKB factor (nuclear factor kappa B) family, are involved in the molecular alterations of the signaling of that factor, with the *NFKB1* (nuclear factor kappa B subunit 1) gene being one of the most relevant, due to the active p50 subunit, encoded by it, being involved in the regulation of a wide variety of inflammatory responses. The *NFKB1* gene, located at chromosomal position 4q24, consisting of 24 exons covering 156 kb, was detected in various types of cells that express, after a series of stimuli, cytokines, chemokines, growth factors, cell adhesion molecules, and some proteins of acute phase in various disease states. Inappropriate activation has been associated with many processes in chronic inflammatory diseases, including inflammatory bowel diseases, especially in NEC^4,7,10,15-17,22,23,28^.

A functional four-base pair insertion/deletion (ATTG) polymorphism in the promoter region of the *NFKB1* gene (-94ins/delATTG) can affect the activity of this region, particularly after stimulation of the innate immune system, destroying the factor binding site transcription and resulting in differential expression of the precursor protein (p105) of the p50 subunit. The variant allele, containing the deletion (del ATTG), produces lower levels of transcription of the p50 subunit, and this affects the availability of the anti-inflammatory homodimer p50/p50 and the pro-inflammatory heterodimer p50/p65. However, the combined effect of relatively low levels of p50/p50 and p50/p65 will be pro-inflammatory, as low levels of p50 will intuitively affect the concentration of p50/p50 more, as it takes two p50 subunits for its composition than the concentration of p50/p65, which requires only one p50 subunit^21,28^.

Abnormalities in the different stages of the intestinal maturation process compromise it by inducing metabolic changes in gene expression and, consequently, in the phenotype. Among the genetic alterations associated with NEC, the -94ins/delATTG polymorphism in the promoter region of the *NFKB1* gene leads to unregulated activation of the NFKB protein, contributing to the development of NEC, due to an increase in the inherent pro-inflammatory state of the premature intestine^15-17,21-23,28^.

The objective of this research was to determine the prevalence of the -94ins/delATTG polymorphism in the *NFKB1* gene in neonates with and without NEC.

## METHODS

This is a case-control study, in which 75 preterm (PTNB) and full-term (FTNB) newborns with and without a diagnosis of NEC, admitted to the Institution’s Neonatal Units, from June 2020 to November 2021, were included.

PTNB/FTNB diagnosed with NEC, based on modified Bell staging^13,20^, were included.

The exclusion criteria were as follows: NB from both groups with clinical or laboratory suspicion of congenital infection; the presence of sepsis and/or meningitis; congenital malformation of the cardiac, digestive, renal, and/or respiratory systems; maternal drug addiction; mothers with any STORCH group infection (syphilis, toxoplasmosis, rubella, cytomegalovirus, herpes) during pregnancy or with human immunodeficiency virus seropositivity; maternal use of opiates or respiratory depressant drugs in the peripartum period; and those whose parents or guardians did not agree to participate in the research.

The NB from both groups were classified, according to gestational age, as post-term NB (≥42 weeks), term NB (38–41 weeks and 6 days), borderline PTNB (36–37 weeks and 6 days), moderate PTNB (31–35 weeks and 6 days), and extreme PTNB (22–30 weeks and 6 days)^3,32^. NB with a birth weight of <2500 g were classified as low weight NB (LWNB), with the following subdivisions: very low weight NB (VLWNB) <1500 g and extreme low weight NB (ELWNB) <1000 g^31^. The entire sample consisted of patients of the same skin color and from the same geographic area (State of São Paulo, Brazil).

### Molecular Analysis

Genomic DNA was extracted from peripheral venous blood leukocytes using the GE Illustra Blood Genomicprep Mini Spin Kit™ Extraction Kit (GE Healthcare UK Limited) according to the manufacturer’s protocol and procedure performed in the Education Sector and Research at the Institution’s Macroscopy Laboratory. The extracted DNA was stored at 4°C for 24 h before being frozen in a -20°C freezer.

To detect the -94ins/delATTG polymorphism, the genomic DNA fragment, which covers the polymorphism region in the promoter region of the NFKB1^27^ gene, was amplified by the polymerase chain reaction (PCR) technique with a PCR reagent kit — OneTaq^®^ Hot Start Quick-Load^®^ 2X Master Mix with Standard Buffer (New England Biolab™) — in a final volume of 25 μl, with previously published^28^ primers, and further digested by the restriction fragment length polymorphism (RFLP) technique, with 10U of PflMI enzyme (New England Biolabs™), at 37°C/1 h, with subsequent inactivation at 65°C/20’.

During the deletion of the 4 base pairs (bp), ATTG is present in both alleles (polymorphic or del/del homozygous sample), and the PCR product is not digested by the PflMI enzyme because there is no recognition of the restriction enzyme site that shows only a 427 bp fragment. In the absence of the polymorphism in both alleles (wild homozygote or ins/ins), the restriction site of the PflMI enzyme is recognized and the PCR product is digested into two fragments of 392 and 39 bp (431 bp). In the heterozygous sample, the polymorphism is present in only one allele; therefore, the PCR product is composed of three fragments: of 427 bp (polymorphic allele with the deletion) and of 392 and 39 bp (wild-type allele).

Products from each of the PCR/RFLP reactions were added to FlashGel™ Loading Dye 5x running head dye and electrophoresed on a 2.2% agarose gel cassette on the FlashGel™ DNA System to confirm their success, and all the gels were photo-documented by the FlashGel™ Camera (Lonza Group Ltd, Muenchensteinerstrasse 38 CH-4002 Basel, Switzerland).

To avoid biases in molecular investigation and the final results, all DNA samples were analyzed without the knowledge of the information obtained from each patient in the study.

### Ethical Aspects

The ethics and research protocol were approved by the Research Ethics Committee (FAMERP, São Paulo, Brazil) (# 3.380.405/2019). Before starting any procedure, the Informed Consent Form was obtained from all parents or guardians.

### Statistical Analysis

The results were previously submitted to descriptive statistics to determine normality. For independent samples when they are normally distributed, the unpaired t-test and the Mann-Whitney U test are applied for samples with non-normal distribution. When applicable, the chi-square or Fisher’s exact tests were used to compare the variables and odds ratio (OR), with a 95% confidence interval (95%CI), to study the OR of the event occurring. The level of significance was set at 5%. The results were expressed as percentage (%), mean (M), median (Median), and standard deviation (SD). Statistical tests were performed using the GraphPad InStat version 3.00 program (GraphPad Software Inc, San Diego, California, USA, www.graphpad.com).

## RESULTS

The total sample consisted of 75 NB: 25 (33%) were diagnosed with NEC, making up the case group, and 50 (67%) were included in the control group.

### Demographic Characteristics

Considering the demographic data of the 25 NB in the case group and of the 50 NB in the control group, male gender was predominant in both groups (52% and 62%), without statistical significance (p=0.4613). Regarding the classification of the NB, according to gestational age, in both groups, there was a higher prevalence of moderate and extreme PTNB, making up a total of 80% in the case group and 88% in the control group. One FTNB (4%) presented a clinical and radiological picture of NEC, thus being included in the case group. There were no cases of post-term NB in either group. The statistical analysis for this demographic variable was not statistically significant (p=0.3036). LWNB and ELWNB were the most prevalent in the case group (78%), and very low weight and extremely low weight were the most prevalent in the control group (81%). The difference between both groups was not statistically significant (p=0.1073). As for the type of delivery, both groups had a higher prevalence of surgical delivery, but without statistical significance (p=1.000). Likewise, in relation to the type of birth, both groups had a higher prevalence of single births, without statistical significance (p=0.3588) (Table 1).

**Table 1.** Demographic characteristics between the case and control groups (%).

Variables	Cases n=25 (%)	Controls n=50 (%)	p-value
Gender			
Male	13 (52)	31 (62)	0.1598*
Female	12 (48)	19 (38)	
NB Classification – GA			
Post-term NB	0 (0)	0 (0)	0.3036^†^
Full-term NB	1 (4)	4 (8)	
Borderline PTNB	4 (16)	2 (4)	
Moderate PTNB	10 (40)	20 (40)	
Extreme PTNB	10 (40)	24 (48)	
Type of Delivery			
Natural	6 (24)	13 (26)	1.0000*
Surgical	19 (76)	37 (74)	
Type of Birth			
Single	22 (88)	38 (76)	0.3588^*^
Multiple (twin)	3 (12)	12 (24)	
NB Classification – LW (<2500 g)	n=18 (%)	n=43 (%)	
LW (≥1500 g and <2500 g)	7 (39)	8 (19)	0.1073^††^
Very LW (<1500 g)	4 (22)	21 (49)	
Extreme LW (<1000 g)	7 (39)	14 (32)	

*Fisher’s exact test; ^†^Chi-square test: 3.635; df-3; ^††^Chi-square test: 4.464; df-2; df: degrees of freedom; n: number; GA: gestational age; NB: new born; PTNB: pre-term new born; LW: low weight.

### Clinical Characteristics of the Case and Control Groups

Regarding clinical characteristics, the mean gestational age between the case and control groups was 31.6 and 31 weeks, respectively, with no statistically significant difference (p=0.4891). The mean weight was 1843.5 and 1561.1 g for the case and control groups, respectively, with no statistical significance (p=0.2471). As for the Apgar score in the 1^st^ minute, the mean value was 6.2 for the case group and 5.2 for the control group, and in the 5^th^ minute, the mean values were, respectively, 8.4 and 7.6, with no statistically significant difference (p=0.05090) (Table 2).

**Table 2.** Clinical characteristics between the case and control groups.

Variables	Cases	Controls	p-value^*^
GA (weeks)			
Mean (±SD)	31.6 (±3.8)	31.0 (±3.8)	0.4891
Minimum	24	25	
Maximum	38	39	
Median	32	31	
Weight (g)			
Mean (±SD)	1843.5 (±1007.9)	1561.1 (±927.9)	0.2471
Minimum	660	630	
Maximum	4034	4130	
Median	1700	1265	
Apgar 1^st^ minute			
Mean (±SD)	6.2 (±2.5)	5.4 (±2.2)	0.1751
Minimum	0	0	
Maximum	9	9	
Median	6	5	
Apgar 5^th^ minute			
Mean (±SD)	8.4 (±1.7)	7.6 (±2.0)	0.0590
Minimum	2	0	
Maximum	10	10	
Median	9	8	

*Mann-Whitney U test; SD: standard deviation; GA: gestational age; Apgar: newborn assessment score at 1 and 5 min after birth.

### Clinical Characteristics of the Cases Group

According to modified Bell staging^13,20^, 72% of the cases were classified as II-A and III-B together. Among the seven patients who required surgical treatment, five (71%) were staged as III-B. Among the three deaths, two (67%) patients with stage III-B received only clinical treatment due to hemodynamic instability, therefore, not allowing the surgical procedure, and one (33%) patient with stage II-B underwent surgical treatment. Clinical treatment was successful in 72% of cases, and hospital discharge was achieved in 88% of patients with NEC. To assess the gestational age at the time of diagnosis of NEC, the age at birth, obtained by somatic Capurro, of the 25 NB in the case group was corrected, obtaining the results in weeks, as shown in Table 3.

**Table 3.** Clinical characteristics of the case group at the diagnosis of NEC.

Characteristics	Case Group
Corrected GA (weeks)	
Mean (±SD)	34.56 (±4.0).
Minimum	27
Maximum	41
Median	36
NEC Staging	n=25 (%)
II-A	9 (36)
II-B	7 (28)
III-B	9 (36)
Treatment	
Exclusively clinical	16 (64)
Surgical	9 (36)
Outcome	
Hospital discharge	22 (88)
Death	3 (12)

GA: gestational age; SD: standard deviation; NEC: necrotizing enterocolitis; n: number.

### Molecular Results

Table 4 shows the genotypic and allelic results found in the case and control groups related to the -94ins/delATTG polymorphism in the *NFKB1* gene.

**Table 4.** Genotypic and allelic relationship for the -94ins/delATTG polymorphism in the *NFKB1* gene between the case and control groups.

Gene/Polymorphism	Genotypes/Alleles	Cases n=25 (%) Alleles n=50 (%)	Controls n=50 (%) Alleles n=100 (%)	OR (95%CI)	p-value
*NFKB1*/-94ins/delATTG	ins/ins	25 (100)	75 (100)	NA	–
	ins/del	0 (0)	0 (0)		
	del/del	0 (0)	0 (0)		
	ins	50 (100)	100 (100)	NA	–
	del	0 (0)	0 (0)		

n: number; NA: not analyzed; OR: odds ratio; CI: confidence interval.

The -94ins/delATTG polymorphism, in the promoter region of the *NFKB1* gene, was not identified in all 150 alleles analyzed (100%).

## DISCUSSION

The molecular basis of NEC is still largely unknown and worthy of scientific investigation. Recent advances in genetic research have allowed a new approach to elucidate the pathophysiology of this condition.

The NFKB protein is an important transcription factor that regulates the expression of several inflammatory genes in response to the TLR, to receptors similar to the nucleotide ligand oligomerization domain (NOD-like receptors (nucleotide oligomerization domain receptors)), and the activation of the interleukin pathway. NFKB is regulated, in part, by the inhibitory protein IκB that binds to and sequesters NFKB in the cytoplasm, preventing its translocation to the nucleus to induce the expression of various inflammatory genes. Immature intestinal epithelial cells, however, have lower levels of IKB, resulting in decreased NFKB inhibition, leading to an inherent pro-inflammatory state of the premature intestine. Thus, the unregulated activation of the NFKB protein contributes to the development of NEC^15-17,21-23,28^.

So far, only one study has analyzed the NFKB1 (-94delATTG) variant in PTNBs with and without NEC^28^, as performed in the present investigation. In the reference study of the total sample, 15 PTNB with NEC were analyzed (5.6%), all with very low birth weight (100%), with a mean gestational age at birth of 25.9 weeks, 67% of Afro-Americans and male predominance (53%), with statistical significance^28^. This study differs from the present one in all these demographic and statistical data, except for the predominance of males, which also occurred in both groups.

There was no significant difference among all the variables analyzed in this study. Despite this, the male gender was more prevalent in both groups, as mentioned. There are trends in gender-specific differences in outcomes in extreme preterms^12^ and also in heterozygous loci related to infection protection that are located on chromosome X^24^. The male gender has fewer of these loci than the female gender due to the presence of a single X chromosome and is therefore more susceptible to the consequences inherent to infectious/inflammatory processes^24^.

The incidence of NEC varies from 1% to 8% of all NB admitted to the neonatal ICU, with high mortality ranging from 10% to 50%. In PTNB weighing less than 1500 g, the incidence of NEC varies from 4% to 13%. Those born at or near term can also be affected in 5–10% of classic cases of the disease^2,8,35^. The present study differs from the reference study^28^ and from the literature^2,8,35^. The present study differs from the reference study^28^because, in this study, 33% of the total sample had NEC, with 61% of PTNB weighing less than 1500 g. These differences may be due to the composition of the sample, ethnicity, geographic area, methodologies, etc. The lethality rate (12%) and the prevalence of NTR with NEC (4%) are in agreement with the literature^2,8,35^ but, even though the reference study has presented a lethality rate (33%), within the range described in the literature, it was higher than in the present study, probably due to the inclusion in this reference study of only extreme preterm infants, of very low weight, with a mean gestational age of 25.9 weeks^28^.

In this reference study, the PTNB developed NEC at 31 days of life (~4 weeks), on average, with no reference to the corrected gestational age at the time of diagnosis^28^. In the literature, the described peak of the distribution of age at the onset of NEC occurs between 29 and 31 weeks of corrected gestational age^25,29,33^. In the present study, the distribution of NEC development was between 27 and 41 weeks of corrected gestational age, with a peak at 36 weeks. There is a need for more investigations with a larger sample for possible explanations related to this later age peak for the onset of NEC found in the present study.

The harmony between the diagnosis and management of NEC should be based on clinical and radiological signs and symptoms in the abdomen, paying attention to frequency peaks according to the corrected gestational age^33^. Once NEC is suspected, the NB must undergo a regular routine of abdominal radiography that aims to monitor the progression of the disease and guide clinical management. The timing of follow-up abdominal radiographs depends on the severity of the NEC and ranges from 6 to 24 h. This follow-up helps to indicate the need for surgical intervention^1,9,18,19^. The protocol was rigorously followed in the conduct of the cases in this study.

Radiological signs of NEC include localized bowel distension, generalized bowel distension, bowel wall thickening, intestinal pneumatosis, air in the hepatic portal venous system, and pneumoperitoneum. However, even with these predictors, diagnosing NEC is a very difficult task for physicians^1,9,18,19^. Studies have been carried out to better understand the etiology of NEC and ways to prevent its progression; however, few have resulted in significant results that have led to changes in clinical practice. Some NBs present the disease so acutely and severely that the morbidity or mortality cannot be avoided despite treatment. The identification of early signs of the disease, both clinical and imaging, can allow for more accurate diagnoses and treatments^5,6,17,26,33^.

Currently, genetic tests are being carried out to investigate the most diverse diseases, not only because they are considered noninvasive, but also because of their high sensitivity and specificity for molecular studies, as performed in the present study and in accordance with the reference study^28^. In this reference study, all very low birth weight PTNBs in the case group (100%) and 65% in the control group presented the variant NFKB1 allele (-94delATTG), unlike the present study, which, in the entire sample, consisted of 75 NB and in the molecular analysis of 150 alleles, the variant del allele was not determined.

To assess the results of genetic association, it is important that the research groups have the same ethnicity (or skin color) and the same geographic origin, as the genetic bases of diseases, such as the polymorphic configuration, can differ between different regions and populations^34^, which was considered in the present study, because despite the intense Brazilian miscegenation, patients and controls with apparent similarity in skin color and from the same geographic area were included. This difference in the molecular result, in relation to the reference study^28^, may be due to the fact that it included, as previously reported, only extremely low-weight PTNB, Caucasian, and African-American preterm infants, in addition to a single exclusion criterion (congenital malformations). In the present study, there were several exclusion criteria, including that of the reference study, all of which were strictly followed.

Although the results obtained by the molecular approach performed in the present study did not determine the variant allele, they should be interpreted with caution and need to be corroborated by independent and/or multicenter studies to determine the real prevalence of the -94ins/delATTG polymorphism in the *NFKB1* gene and its association with NEC in the general population and also in the Brazilian population.

Greater knowledge of this molecular relationship is important not only for understanding the physiology and physiopathogenesis of NEC but also for discovering its genetic causes. This will provide new perspectives and guide humanized and personalized protocols through therapeutic schemes and different technologies, which provide early diagnosis and interventions, even in the face of these genetic alterations, in addition to stimulating the continuity of scientific research.

## CONCLUSIONS

The absence of the -94ins/delATTG polymorphism in the *NFKB1* gene in NB with and without NEC does not rule out the possibility of the existence of alterations in this and/or other genes in NB with this condition, which reinforces the need for further research.
